# Horner's Syndrome as a Rare Complication of Thyroidectomy With Central Neck Dissection: A Case Report and Review of Potential Mechanisms

**DOI:** 10.7759/cureus.78841

**Published:** 2025-02-11

**Authors:** Merve Tok, Reyhan Taskin, Zehra Kara, Kenan Cetin

**Affiliations:** 1 General Surgery, Çanakkale Onsekiz Mart University, Faculty of Medicine, Çanakkale, TUR

**Keywords:** central neck dissection, horner syndrome, miosis, ptosis, thyroid surgery

## Abstract

Horner's syndrome (HS) is characterized by miosis, ptosis, enophthalmos, anhidrosis, and vascular dilation on one side of the face, resulting from an interruption in the oculosympathetic pathway. Here, we present a 55-year-old female patient diagnosed with thyroid carcinoma metastasis via fine-needle aspiration biopsy of the right supraclavicular lymph node. She underwent total thyroidectomy and right central lymph node dissection. On the first postoperative day, the patient developed right-sided miosis and upper eyelid ptosis, leading to a diagnosis of HS. Although this rare complication following thyroid surgery has been reported, its exact mechanism remains unclear. We attribute this occurrence to cervical sympathetic chain injury during aggressive central dissection.

## Introduction

Horner's syndrome (HS), first identified by Johann Friedrich Horner in 1869, presents with characteristic symptoms, including miosis, ipsilateral ptosis, enophthalmos, facial anhidrosis, and vascular dilation on one side of the face [1,2]. These manifestations stem from disruption of the oculosympathetic pathway, often due to lesions affecting the cervical sympathetic chain. HS is well-documented as a result of compression by large benign or malignant goiters. However, cases associated with thyroidectomy are rare [3], with an incidence of less than 0.2-0.3% among patients undergoing this procedure [3,4].

In a study by Cozzaglio et al. involving 495 thyroidectomy patients, only one case of postoperative HS was identified, highlighting its rarity [3]. Given the close anatomical relationship of the oculosympathetic pathway with the thyroid gland, this structure is inherently at risk during surgical intervention. Most etiologies involve compression or injury to the cervical plexus, and manipulation of the parapharyngeal space can further predispose patients to HS [5,6]. Herein, we report a case of HS following total thyroidectomy and right central lymph node dissection in a 55-year-old female patient with papillary thyroid carcinoma, emphasizing the importance of awareness of this rare complication.

## Case presentation

A 55-year-old female patient was referred to our clinic by the medical oncology team for total thyroidectomy and neck dissection. Her medical history included a diagnosis of HER2-positive, HR-negative invasive breast carcinoma, for which she had previously received neoadjuvant therapy, followed by left mastectomy with axillary dissection and subsequent radiotherapy at an external center. Preoperative PET-CT revealed a right supraclavicular lymph node approximately 2 cm in size near the thyroid gland, with punctate calcifications and an SUVmax of 6.4. Excision of this lymph node was performed concurrently with her mastectomy, and pathology confirmed thyroglobulin-positive thyroid carcinoma metastasis.

**Figure 1:**
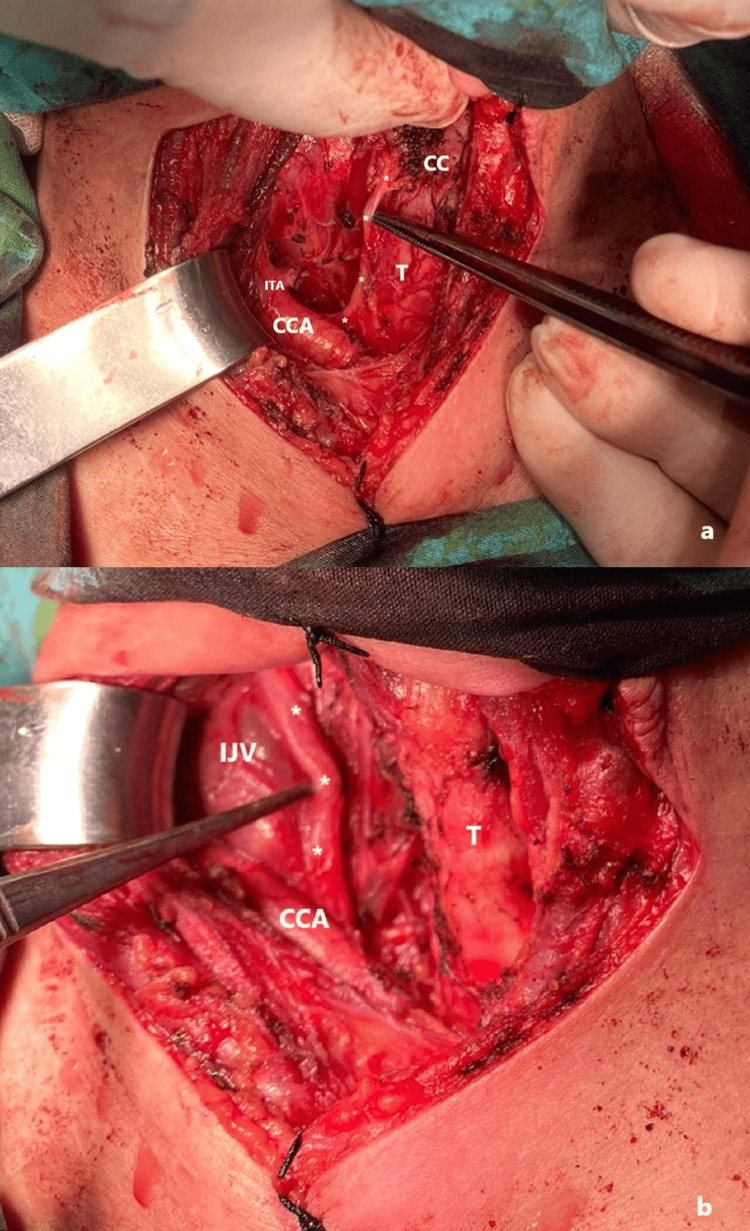
Total thyroidectomy with central lymph node dissection. Right central compartment with lymph nodes removed * RLN in panel (a) and the vagus nerve in panel (b) T: trachea, CC: cricoid cartilage, CCA: common carotid artery, ITA: inferior thyroid artery (ligated proximally), IJV: internal jugular vein, RLN: recurrent laryngeal nerve

Thyroid ultrasound identified several heterogeneous, isoechoic solid to semi-solid nodules in both lobes and the isthmus, with the largest nodule located in the lower right lobe, measuring 25 x 18 mm, and another in the lower left lobe, measuring 14 x 7 mm. Some nodules exhibited internal-peripheral vascularity, with microcystic areas and millimetric calcifications. Consequently, the patient underwent total thyroidectomy and unilateral therapeutic central lymph node dissection (Figure 1). The thyroid gland was first mobilized via medial retraction following careful ligation of the superior thyroid vessels. The right central compartment was meticulously dissected, and lymph nodes were removed en bloc while preserving the recurrent laryngeal nerve (RLN) and parathyroid glands. The inferior thyroid artery was ligated proximally near its origin. However, due to extensive metastatic involvement, aggressive dissection in this area increased the risk of injury to the cervical sympathetic chain. Additionally, retraction of the carotid sheath and manipulation of adjacent structures may have contributed to traction injury or direct nerve trauma, ultimately leading to HS.

**Figure 2:**
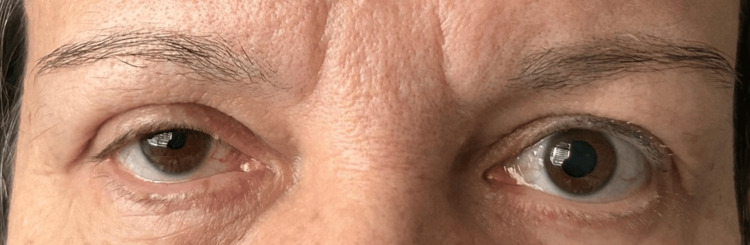
Right-sided miosis and eyelid ptosis observed postoperatively

In the immediate postoperative period, the patient reported redness and a stinging sensation in her right eye (Figure 2). Physical examination revealed right-sided miosis, ptosis, inverse ptosis, delayed dilation of the right pupil, and anisocoria, though anhidrosis was absent. Ophthalmology and neurology consultations were obtained, including orbital and neck CT and orbital, brain, and cervical spine, revealing no acute pathological findings.

## Discussion

Swiss ophthalmologist Johann Friedrich Horner first described HS in 1869 [1,2]. It is characterized by miosis, ptosis, enophthalmos, anhidrosis, and vascular dilation on one side of the face due to damage to the ipsilateral cervical sympathetic chain. Common causes of HS include cerebrovascular injury, mediastinal tumors, neck trauma, and iatrogenic injuries during neck surgery [7]. Although extremely rare, HS can occur as a complication of thyroidectomy [2,6]. In our case, as in similar reports, vascular symptoms such as ipsilateral anhidrosis and cutaneous vascular dilation were notably absent.

Recovery rates vary among patients who develop HS after thyroidectomy. Approximately 70% of cases do not fully resolve, while the remainder recover from five days to 15 months postoperatively. In cases where HS is caused by sympathetic plexus injury, spontaneous resolution is more common [6].

Potential mechanisms leading to HS following thyroidectomy include stretching the sympathetic chain due to lateral retraction of the carotid sheath, which may cause a neuropraxia-type injury [7]. Postoperative hematoma formation can compress the sympathetic chain, contributing to HS. Additionally, ligation of the inferior thyroid artery, which supplies blood to the cervical sympathetic chain, may lead to ischemia; Solomon et al. suggested that ligation of this artery or its branches could result in HS [8]. The anatomical proximity of the sympathetic chain to the inferior thyroid artery further increases the risk of injury during thyroidectomy. Disruption during RLN identification may inadvertently affect the connection between the RLN and sympathetic innervation [5,7].

The oculosympathetic pathway consists of three distinct neurons, and any lesion along this pathway can result in HS. The first-order neuron originates in the hypothalamus, synapsing at the C8-T2 level of the spinal cord. Second-order preganglionic fibers exit the spinal cord at the T1 level, entering the cervical sympathetic chain and ascending past the lung apex to synapse at the superior cervical ganglion near the carotid bifurcation (C3-4). Third-order neuron fibers follow the carotid artery system, progressing through the cavernous sinus and superior orbital fissure to reach the orbit. In our patient, right-sided blepharoptosis and miosis developed without accompanying anhidrosis, suggesting a lesion distal to the superior cervical ganglion or after the branching of sudomotor fibers [4].

Additionally, retractors used to expose the thyroid gland may inadvertently cause blunt or direct injury to surrounding tissues, including the carotid artery and adjacent sympathetic nerves. In some individuals, oculosympathetic fibers may adhere to the thyroid gland, or communication branches may exist between cervical sympathetic nerves and the RLN, increasing the likelihood of unintentional injury to oculosympathetic fibers. Furthermore, in our case (Figure 1), proximal ligation of the inferior thyroid artery posed a risk of damaging adjacent sympathetic nerves. Another factor contributing to HS may be thermal injury from thermodynamic devices used in surgery [9]. In 2019, Sapalidis et al. noted an increased incidence of postoperative HS following more extensive procedures, such as lateral neck lymph node dissection [10].

HS should be distinguished from other conditions causing ptosis and pupillary abnormalities, such as third cranial nerve palsy, cavernous sinus syndrome, and carotid artery dissection. Accurate diagnosis requires a detailed history, clinical examination, and imaging studies like brain and cervical spine MRI or CT angiography. To minimize the risk during thyroidectomy and neck dissection, surgeons should use gentle retraction, avoid excessive traction on the carotid sheath, preserve the inferior thyroid artery when possible, and protect adjacent neurovascular structures. Preoperative imaging can help identify anatomical variations and ensure a safer surgical approach.

Postoperative management depends on the severity and cause of injury. Mild cases may improve with observation and symptomatic treatment, while pharmacologic interventions like apraclonidine eye drops can temporarily relieve ptosis and miosis. Long-term neurological follow-up is essential, and physical therapy may benefit cases with associated neck trauma or nerve dysfunction. Understanding the mechanisms of injury and recognizing anatomical variations can help reduce the incidence of this rare but significant complication.

## Conclusions

HS is a rare complication of thyroidectomy. Awareness of the underlying causes and anatomical variations associated with this syndrome can aid in minimizing its occurrence. Surgical teams should be mindful of this uncommon yet significant complication. The intricate neurovascular structures surrounding the thyroid gland make it particularly vulnerable to accidental injury during surgery; thus, meticulous dissection is essential to protect peripheral nerves and blood vessels. This careful approach minimizes damage to surrounding tissues, promotes faster postoperative recovery, reduces patient discomfort, and achieves optimal cosmetic outcomes. The close anatomical relationship between the thyroid gland, adipose tissue, and cervical sympathetic chain underscores the importance of precision in thyroidectomy and selective neck dissection.
